# What is the authentic underlying reason of childbirth worries in Turkish population?: An observational study

**DOI:** 10.1097/MD.0000000000039306

**Published:** 2024-08-16

**Authors:** Nükhet Kaçar, Rahime Bedir Findik, Özlem Moraloğlu Tekin

**Affiliations:** a Ministry of Health, Ankara City Hospital – Maternity Hospital, Ankara, Turkey.

**Keywords:** fear, midwifery, parturition, pregnant women

## Abstract

This study aims to determine women’s childbirth worries during antenatal. The research was carried out with 532 pregnant women in the antenatal clinic in Turkey as an observational study. Sociodemographic characteristics and scores of the Oxford Worries about Labor Scale of pregnant women were evaluated quantitatively. In addition, the answers given by the pregnant women to the open-ended question were themed. Although working status and receiving antenatal education reduce the fear of childbirth, birth scenes/stories on TV or social media, birth stories in the pregnant women’s friends/family, being stressed in daily life, and dysmenorrhea increase the worries about childbirth (WaC). In addition, primiparas experience more WaC than multiparas. The reasons for WaC in pregnant women were classified as birth pain, artificial pain, cesarean section/receiving anesthesia, intervention/examination, pandemic, people’s thoughts/experiences, birth process/insufficiency in birth, hospital/staff, fears about the baby, complications/death, and ignorance of the birth process. The results of this study reveal that WaC is a pivotal issue for pregnant women, for which managing the labor process, labor pain and labor fear is important. The stipulation of support for pregnant women is essential to enhance labor outcomes.

## 1. Introduction

Being pregnant and giving birth is a peerless experience for each woman. The labor process has multidimensional characteristic features which can be affected by anxiety, apprehension, fears, trauma, exploitation, insufficient social support, economic issues, attitudes toward giving birth, expectations, cultural structures, the quality of the delivery room, and the care given by a midwife or doctor. Worries about childbirth (WaC) are one of the dimensions of labor (WaC).^[1–5]^ Although most women give birth as a spectacular experience, this can also be a frightening experience for some women.^[3,6]^

WaC is a common issue, which affects the health indicators of pregnancy, childbirth, and postpartum. In addition, its prevalence changes among countries.^[1–6]^ WaC also causes women to want a cesarean section in some countries which are even proven safe for maternity care.^[3]^

The research area of WaC is comprehensive and complicated. Many scales are developed to evaluate WaC, and many methods are used to reduce WaC. The prevalence and reasons of WaC are investigated and the level of WaC is measured by using any scales in the literature. In addition, there are many studies about using non-pharmacological methods such as hypnotherapy, relaxation techniques, psychoeducation, mindfulness-based birth education, and musicotherapy to reduce the WaC and enhance birth outcomes.^[3,6–8]^

The prevalence and reasons of WaC, and the discrepancy of using methods to reduce the WaC prevent making a consensus on the research. For this reason, it is indicated that WaC is necessary to evaluate widely.^[3]^

The aims of our research are (first) to measure the level of WaC by using the Oxford Worries about Labor Scale (OWLS), (second) to evaluate reasons for WaC, (third) to research the predisposing risk factors of WaC, and (fourth) to present open-ended finds about WaC.

## 2. Methods

### 2.1. Research sample and design

This study was conducted as an observational study.

The pregnant women (n = 700), who are 18 to 35 years old, have no obstetric complications, are on any gestational week and go to antenatal polyclinic on 15 September to 15 December 2021 were invited by the researchers. The pregnant women who agreed to attend the study received permission from an informed consent form. One hundred twelve of them did not want to attend the study. Eighteen of them (n = 588) wanted to leave the study. Thirty eight of them (n = 570) did not complete the questionnaire. Therefore, we could include 532 pregnant women in the study.

In the first stage, the pregnant women accepted to attend the study (n = 588) filled out the descriptive questionnaire which is constituted by researchers in line with the literature and does not have any validity and reliability. In this stage, 10 pregnant women wanted to leave the study.

In the second stage, the WaC of pregnant (n = 578) were evaluated by using OWLS.

In the third stage, (n = 570) pregnant women’s thoughts about WaC and the predisposing factors caused by WaC are evaluated by open-ended questions. 38 pregnant women were not included in this stage as they answered incomprehensibly and incompletely.

Five hundred thirty two pregnant women, who continued the study, and filled out or answered the questions comprehensibly and completely, were included in the analysis.

Five hundred thirty two pregnant women were analyzed in line with the literature.^[9,10]^ The other research about WaC analyzed 544 pregnant women^[2]^ and 401 pregnant women^[8]^ respectively.

### 2.2. Variables and registration

The research is an observational study, for which it is registered on the clinical trial system with NCT05131503 number in June 16, 2021.

A priori power analysis with G*Power (3.9.1.4) was done by the researcher and it indicated that a sample size of 479 would be sufficient to detect a significant small effect of WaC, assuming Odds Ratio = 1.4, alpha of 0.05 and Power of 0.90, by taking as references studies.^[2,8]^

### 2.3. Instruments

This scale was constituted by Redshaw et al,^[11]^ which has 10 questions as a 4-point Likert scale. This scale can be used on prepartum, intrapartum and postpartum. The expressions of this scale are “I was very worried;” “I was quite worried;” “I was not quite worried;” and “I was not worried at all.” The scale’s total point changes between min = 10 and max = 40. As the point in the scale increases, the WaC of women decreases. Turkish validity and reliability of this scale were made by Erkal Aksoy and Gülsün Özentürk.^[12]^ In this stage, 8 pregnant women wanted to leave the study.

### 2.4. Statistical analysis

Descriptive parameters were analyzed by using frequency, percent, minimum, maximum, and standard deviation statistics. Levene test was used for the equality of variances and the independent sample *T* test was used for comparing two groups. Test of homogeneity of variances Levene and 1-way ANOVA test were used for comparing three groups. When the significant difference was determined for three groups, if the variances were homogeneous, Tukey was used, if it was not, Games–Howell was used. The correlation among the statements was analyzed by Pearson Correlation.

### 2.5. Ethics approval

The ethical approval was received from Ankara City Hospital 2 Numbered Clinical Research Ethics Committee on June 16, 2021, with Decision No. E2-21-369. In addition, the pregnant women who agreed to attend the study received permission from an informed consent form.

## 3. Results

Mothers who have any antenatal class/education and work had fewer labor worries. The pregnant women who agreed with the statements of “the scenes,” “the stories” and “the experiences” had more labor worries. The pregnant women, who felt stressed in their casual life had more labor worries. Besides women with dysmenorrhea had more labor worries (Table 1).

**Table 1:** The comparison of OWLS points of two expressions/features.

Parameters	Percentage (%)	OWLS points	*t* *	*P*
Age				
18 to 29 years old	67.3	23.17 ± 6.817	−1.540	.124
30 to 35 years old	32.7	24.14 ± 6.819		
Working status				
Yes^a^	18.2	26.10 ± 7.107	4.244	**.000**
No^b^	81.8	22.90 ± 6.631		**a > b**
Antenatal class/education				
Yes^a^	24.1	25.48 ± 6.626	3.852	**.000**
No^b^	75.9	22.85 ± 6.774		**a > b**
Do the scenes of childbearing on TV affect your thoughts on childbirth?				
Yes^a^	20.7	21.73 ± 6.519	−3.056	**.002**
No^b^	79.3	23.94 ± 6.838		**a < b**
Do the stories of childbearing on social media affect my thoughts on childbirth?				
Yes^a^	33.3	22.30 ± 6.280	−2.847	**.005**
No^b^	66.7	24.08 ± 7.017		**a < b**
Do the experiences of childbearing of my family or friends affect your thoughts on childbirth?				
Yes	45.5	21.92 ± 24.79	−5.005	**.000**
No	54.5	24.79 ± 7.075		**a < b**
Are you supported by my family partner or friends during your pregnancy process?				
Yes	88.9	23.51 ± 6.849	.194	.846
No	11.1	23.32 ± 6.694		
I am a stressed person in daily life				
Yes^a^	42.9	22.55 ± 7.009	−2.745	**.006**
No^b^	57.1	24.18 ± 6.612		**a < b**
Have you ever watched a video of a human giving birth?				
Yes	47.0	23.76 ± 6.305	.895	.371
No	53.0	23.24 ± 7.260		
Have you ever watched a video of an animal giving birth?				
Yes	24.8	23.48 ± 6.752	.000	1.000
No	75.2	23.49 ± 6.859		
Pregnancy number				
Primigravida^a^	38.7	22.27 ± 6.977	−3.288	**.001**
Multigravida^b^	61.3	24.25 ± 6.626		**a < b**
Do you think that you have a risky pregnancy?				
Yes	17.3	23.48 ± 6.514	−.010	.992
No	82.7	23.49 ± 6.897		
Do you think that your childbirth needs any intervention?				
Yes	2.3	22.17 ± 5.952	−.676	.499
No	97.7	23.52 ± 6.847		
Do you have a painful menstrual period?				
Yes^a^	58.3	22.96 ± 6.687	−2.085	**.038**
No^b^	41.7	24.21 ± 6.966		**a < b**
Do you use any alternative methods for decreasing pain during your menstrual period?				
Yes	48.5	23.03 ± 6.608	−1.503	.133
No	51.5	23.92 ± 7.010		
Is your sexual life regular?				
Yes	85.5	23.69 ± 6.769	1.706	.089
No	14.5	22.26 ± 7.076		
Does your sexual life have problems?				
Yes	2.4	23.31 ± 7.499	−.095	.925
No	97.6	23.49 ± 6.816		

* Independent sample *t* test, *P* < .005.

There is a statistically significant difference between the points of OWLS and thoughts of pregnant women, who watched the delivery of humans and/or animals. The pregnant women, who evaluated the childbirth of humans as “riveting” had fewer labor worries than those who evaluated it as “frightening.” The pregnant women who evaluated the delivery of the animal as “impressive” had fewer labor worries than those who evaluated it as “painful” (Table 2).

**Table 2:** The comparison of OWLS points of multi-expressions/features.

Parameters	Percentage (%)	OWLS points	*F* *	*P*
Education level				
Primary-Secondary School	27.3	23.28 ± 6.357	1.822	.123
High School	34.6	22.61 ± 6.902		
Associate Degree	17.5	24.16 ± 6.725		
Bachelor’s Degree	18.6	24.68 ± 6.997		
Postgraduate	2.1	24.36 ± 9.542		
Wanting to have a baby				
Both partners want	96.4	23.57 ± 6.829	1.456	.226
Mother wants but father not	.8	25.75 ± .957		
Father wants but mother not	1.1	21.17 ± 7.600		
Both partners do not want	1.7	19.44 ± 6.784		
Where have you got information about the pregnancy?				
Experiences/previous pregnancy	3.2	25.94 ± 6.833	1.878	.113
Hospital/family health center/antenatal class	49.2	24.06 ± 7.042		
Family/friends/relatives	20.5	22.66 ± 5.631		
Internet/social media	22.2	22.65 ± 7.234		
Book/journal	4.9	23.27 ± 6.809		
Thoughts on humans giving birth (47%)				
A. frightening/worrisome/terrifying	12.6	22.55 ± 6.145	2.651	.034** **A > C**
B. painful/hurtful/effortful/tough	10.2	22.85 ± 6.574		
C. riveting/interesting/miraculous/amazing	9.4	25.94 ± 6.732		
D. impressive/meaningful//valuable/exciting/cheerful	8.6	23.57 ± 5.451		
E. natural/easy/informative/peaceful	6.2	24.70 ± 5.982		
Thoughts on animals giving birth (24.8%)				
A. frightening/worrisome/terrifying	3.6	21.74 ± 8.225	3.459	.010*** **B < D**
B. painful/hurtful/effortful/tough	4.5	19.71 ± 7.434		
C. riveting/interesting/miraculous/amazing	7.9	24.60 ± 6.455		
D. impressive/meaningful//valuable/exciting/cheerful	5.3	25.46 ± 4.749		
E. natural/easy/informative/peaceful	3.6	24.63 ± 5.679		
Evaluation of the previous childbearing (n = 298) (56%) (244 of pregnant women is nulliparous 44%)				
Well	36.3	24.55 ± 6.788	.737	.480
Middle	10.9	24.02 ± 7.010		
Poor	8.8	23.28 ± 5.671		

* One-way ANOVA, *P* < .005.

** Tukey.

*** Games–Howell.

The outcomes of the Pearson correlation test stated that there is a statistically significant difference between working situation, antenatal education status, the labor stories which are on TV, social media, and of family/friends, feeling stressed, number of pregnancies, having dysmenorrhea, thoughts on the delivery of animal and the points of OWLS. However, there is no statistically significant difference between watching the delivery of a human and/or animal, thoughts on the childbirth of humans and the points of OWLS (Table 3).

**Table 3:** The evaluation of the correlation between OWLS points and of multi-expressions/features.

Parameters	OWLS points	Pearson correlation***	*P*
Working status			
Yes^a^	26.10 ± 7.107	−.181	**.000** *
No^b^	22.90 ± 6.631		**a > b**
Antenatal class/education			
Yes^a^	25.48 ± 6.626	−.165	**.000** *
No^b^	22.85 ± 6.774		**a > b**
Do the scenes of childbearing on TV affect my thoughts on childbirth?			
Yes^a^	21.73 ± 6.519	.132	**.002** *
No^b^	23.94 ± 6.838		**a < b**
Do the stories of childbearing on social media affect your thoughts on childbirth?			
Yes^a^	22.30 ± 6.280	.123	**.005** *
No^b^	24.08 ± 7.017		**a < b**
Do the experiences of childbearing of my family or friends affect your thoughts on childbirth?			
Yes^a^	21.92 ± 24.79	.210	**.000** *
No^b^	24.79 ± 7.075		**a < b**
Are you a stressed person in daily life?			
Yes^a^	22.55 ± 7.009	.118	**.006** *
No^b^	24.18 ± 6.612		**a < b**
Have you ever watched a video of a human giving birth?			
Yes	23.76 ± 6.305	−.039	.375
No	23.24 ± 7.260		
Have you ever watched a video of an animal giving birth?			
Yes	23.48 ± 6.752	.000	1.000
No	23.49 ± 6.859		
Pregnancy number			
Primigravida^a^	22.27 ± 6.977	.141	**.001** *
Multigravida^b^	24.25 ± 6.626		**a < b**
Do you have a painful menstrual period?			
Yes^a^	22.96 ± 6.687	.090	.038**
No^b^	24.21 ± 6.966		**a < b**
What are your thoughts on humans giving birth?			
A. frightening/worrisome/terroristic	22.55 ± 6.145	.122	.055
B. painful/hurtful/effortful/tough	22.85 ± 6.574		
C. riveting/interesting/miraculous/amazing	25.94 ± 6.732		
D. impressive/meaningful//valuable/exciting/cheerful	23.57 ± 5.451		
E. natural/easy/informative/peaceful	24.70 ± 5.982		
What are your thoughts on animals giving birth?			
A. frightening/worrisome/terroristic	21.74 ± 8.22	.232	**.007** *
B. painful/hurtful/effortful/tough	19.71 ± 7.434		
C. riveting/interesting/miraculous/amazing	24.60 ± 6.455		
D. impressive/meaningful//valuable/exciting/cheerful	25.46 ± 4.749		**B < D**
E. natural/easy/informative/peaceful	24.63 ± 5.679		

* Correlation is significant at the < .001 level (2-tailed).

** Correlation is significant at the < .005 level (2-tailed).

*** Pearson correlation.

**Figure 1.**
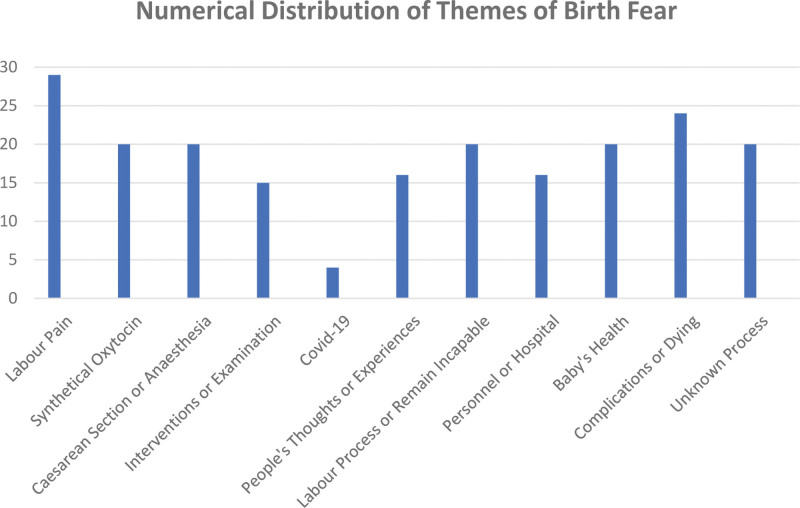
The numerical distribution of themes of birth fear.

Fifteen types of themes (Fig. 1), which consist of the responses to open-ended questions given by pregnant women were identified. The participant was abbreviated as “P” (Table 4).

**Table 4:** The themes related to birth fears of pregnant women.

	Themes	Participants*
1	Labor pain or ache (29)	**P11**, P20, **P27**, **P29**, **P43**, P47, **P49**, P51, **P52**, P59, **P67**, P71, P86, P90, **P102**, P106, P173, **P178**, **P312**, P320, **P331**, P339, P358, **P359**, P364, P374, **P409**, P423, P480
2	Synthetical oxytocin (20)	**P9**, **P27**, P46, P63, P107, P113, P128, P193, P267, **P331**, P333, P335, P337, **P365**, P397, P432, P464, P474, P483, **P488**, P511
3	Caesarean section/anesthesia (20)	P7, **P44**, **P45**, P48, **P52**, **P53**, P64, **P98**, **P160**, P288, P294, **P312**, P342, P347, **P395**, P408, P455, P456, P495, P496
4	Interventions/examination (15)	P6, **P45**, **P49**, **P53**, **P95**, **P98**, **P126**, **P177**, **P201**, P234, **P312**, **P355**, **P365**, **P391**, **P460**
5	COVID-19 (4)	**P3**, P70, P85, **P426**
6	People’s thoughts and experiences (16)	**P3**, P4, P8, **P11**, P32, **P49**, **P50**, **P95**, P131, P136, **P178**, P215, P427, P307, **P395**, P492
7	Labor process/Remain incapable (20)	P2, P5, P15, P25, P28, P36, P115, P160, P166, **P168**, P181, **P204**, P262, **P338**, P352, **P359**, P376, P386, P440, P504
8	Personnel/hospital (16)	**P3**, **P9**, **P45**, **P55**, P84, **P95**, **P98**, **P126**, **P177**, **P279**, **P318**, **P355**, P404, **P409**, P473, **P488**, **P518**
9	About baby (20)	**P3**, **P9**, P16, P21, P40, **P67**, **P49**, **P67**, **P75**, P110, **P168**, P169, P192, P200, **P204**, **P336**, **P338**, **P353**, **P426**, **P520**
10	Complications/dying (24)	**P3**, P14, P24, P26, **P27**, **P34**, **P44**, P54, **P55**, **P67**, **P72**, **P75**, P92, **P102**, P104, **P155**, P165, P194, P305, **P312**, P327, P332, P383, P468
11	Unknowing process (20)	**P29**, P30, **P43**, **P49**, P56, P68, P99, P133, P195, P246, P291, P300, P313, **P336**, **P355**, P390, **P409**, **P460**, **P469**, **P510**

Participants expressing more than one fear are bolded.

Pregnant women’s statements are in Table 5.

**Table 5:** The pregnant women’s statements.

Pregnant women’s statement examples
Theme 1. *Pregnant women’s expressions about their fears as to labor pain.* I fear experiencing labor pain or ache (P20, P47, P51, P59, P71, P86, P90, P106, P173, P320, P339, P358, P364, P374, P423, P480). I am afraid of childbirth because of videos about labor and the people around me and experience labor pain (P11, P178). I am really scared of labor and because of this situation, I feel have hypertension. Also, I am afraid of synthetical oxytocin. If this hospital has epidural labor analgesia, I want to give birth with it (P27). I listened to stories about tough labor and I am afraid. Also, I am worried about if my baby is dying or using a vacuum on my baby’s head. I don’t know if I can take care of my baby. I am afraid what if I am not good (P49).
Theme 2. *Pregnant women’s expressions about induction labor by using medication that is synthetical oxytocin.* I am afraid of labor induction and what if the doctor does not take care of my decisions (P9)? I fear labor induction or I am exposed to synthetical oxytocin (P46, P63, P107, P128, P193, P333, P335, P337, P397, P432, P464, P474, P483, P511). I am afraid of labor induction that’s why I want to give birth with epidural analgesia (P113). I fear labor induction that’s why I am afraid of losing my self-control on my birth process (P488).
Theme 3. *Pregnant women’s expressions about the fear of cesarean section and anesthesia.* I am afraid of experiencing cesarean section (P7, P48, P64, P342, P456). I fear anesthesia (P288, P294, P347). I am afraid of what if I have a brain embolism or cesarean section (P44). I am afraid of experiencing cesarean section, interventions on labor and not concerning by personnel (P45). I am worried about experiencing a cesarean section after its pain (P52). I am terrified of cesarean section. I would love to give birth normally without any interventions. Thus, I read a book about the labor process (P53). I am afraid if my cervical dilatation does not progress that’s why I must be exposed to cesarean section (P359). My first childbirth was a cesarean section. I would like it to be normal, but it was not. I am terrified of cesarean *section and other women’s screams (P395).* I am terrified of the cesarean section that’s why I would like to have a vaginal birth after cesarean (P496).
Theme 4. *Pregnant women’s expressions about the fears as to interventions.* I had so many sutures during my previous childbirth. Thus, I am worried about it (P6). I am terrified of experiencing labor pain, anesthesia and my vaginal opening enlarged (P312). I am afraid of synthetical oxytocin and episiotomy (P365). I am terrified of getting an examination (P95, P201, P391).
Theme 5. *Pregnant women’s expressions about the fears of COVID-19.* I am worried about my baby’s health and experiencing any complications. And I am worried about having COVID-19 and the effects of this illness on my baby. I am afraid of the midwife and doctor how to act to me. I fear birthing stories of people (P3). I am afraid of having COVID-19 (P70, P85). I had COVID-19 so I am afraid of affecting on baby (P426).
Theme 6. *Pregnant women’s expressions about the fears as to people’s thoughts and experiences.* Thoughts or experiences of the people scare me (P4, P8, P131, P136, P215, P427). This is my first pregnancy and I am afraid of the other pregnant women’s approaches (P307). I am afraid of the stories that the people experiencing childbirth are telling (P32, P492).
Theme 7. *Pregnant women’s expressions about the fears as to labor process or remain incapable.* I tired during my pregnancy. I doubt that if I am not able to push my baby and have shortness of breath (P5). I am afraid of what if my labor pain doesn’t start by itself (P166). The reason I have a fright is because my previous childbirth was a negative experience (P181). I am terrified of vaginal birth. Thus, I cannot sleep. I would love to experience a cesarean section (P262). I gained too much weight so I fear that I cannot push my baby (P352). I fear that my cervical dilatation does not progress (P15, P36, P115). I am afraid of giving birth prematurely (P25, P28, P160, P504).
Theme 8. *Pregnant women’s expressions about the fears as to personnel or hospital.* The reason I am worried about the behaviors of personnel is because I was persecuted by personnel in my previous birth (P84). I am afraid of hospitals and personnel. I fear the stories that other people are telling. I am terrified of getting an examination. I want the personnel to support the patient who has vaginismus and to help them. I would like the personnel to be nice to them (P95). I am terrified of exposing to unneedful interventions and misbehaviours because of self-aggrandizement, and not getting information from the personnel (P126). I do not want the doctors and midwives to take control during the labor process. I am afraid of being at the hospital and personnel. I am worried about who assists my childbirth (P279). I am afraid of not taking care of myself. I am ashamed that there are too many personnel in the delivery room, especially men (P318). I am worried that I am assisted in childbirth by midwives without my self-determination. I would like the midwives to help me with childbirth and I want to be active (P518).
Theme 9. *Pregnant women’s expressions about the fears as to baby’s health.* The reason I am worried about my baby’s health is because, in my previous childbirth, my baby went into an incubator (P16). I had a stillbirth that’s why I am terrified. Also, I am worried about labor pain (P67). My baby has spina bifida risk, so I am afraid of it (P110). I am afraid of not being able to breastfeed (P168, P338). My previous baby had cleft lips/palates. For this reason, I am worried about my baby (P353).
Theme 10. *Pregnant women’s expressions about the fears as to complications or death.* What if I bleed during childbirth and what if I am dying? (P14, P34, P327, P468). In my first pregnancy, I had hypertension. I had been at the hospital for two weeks. I am afraid of experiencing this situation again (P24). I am afraid that my placenta is getting old (P26). I fear pieces of my placenta remaining within my womb (P72). In my previous childbirth, my baby had cord entanglement. I am afraid of it (P75). My friend died while she was giving birth. I am afraid of this situation (P103). In my first childbirth, I had so many tears without episiotomy that’s why I had to diet. Thus, I am afraid of childbirth (P104). I fear dying after childbirth (P351).
Theme 11. *Pregnant women’s expressions about the fears as to unknowing labor process.* This is my first pregnancy, and it will be my first childbirth. So, I am worried that I do not know the process of labor (P30, P56, P68, P99, P133, P195, P246, P291, P300, P313, P390, P469, P510). I have anxiety because I do not have any experience with childbirth. I would like to give birth completely naturally. I do not want any interventions such as episiotomy or epidural analgesia in my childbirth (P460).

## 4. Discussion

In our study, there is no statistically significant difference between age, educational level, social support, and WaC, even though working pregnant women had less WaC. It was found that there is a statistically significant difference between being young, having a lower educational level, dissatisfaction with the partner’s support and WaC in the study of Gao et al.^[10]^ It was found that pregnant women, who has higher education levels had more WaC in the study of Qiu et al.^[13]^

It was found that the pregnant women, who took antenatal classes/education, had lower WaC in our study. Similarly, it informed us that as accessibility to information about childbirth increases, the probabilities of WaC decrease in the study of Stoll et al.^[14]^ It was stated that antenatal education decreases the WaC and increases the self-efficacy of mothers.^[15]^ It was shown that the pregnant women, who have higher labor self-efficacy had lower WaC and it was emphasized that antenatal education is needed to reduce WaC and to improve the labor self-efficacy of mothers.^[16]^ The systematic analyses of Striebich et al,^[7]^ which persuaded 15 types of research about WaC showed that the self-efficacy of pregnant women can be strengthened, and the cesarean rates related to WaC can be decreased by taking solo or group psychoeducation sessions. Karabulut et al^[17]^ gave antenatal education to one group of pregnant women, and they compared educated and uneducated pregnant women. A statistically significant difference in acceptance of pregnancy and the level of WaC was found among the two groups. It was stated that the adaptation of pregnancy, self-efficacy, and health literacy increased and WaC in pregnant women decreased by taking antenatal education.^[18]^

The pregnant women, who feel stressed in their casual life had more labor anxiety in our study. Another study stated that stress, worries, depression, and insufficiency of social support were related to the fear of pregnancy.^[1]^ In parallel with our study, a statistically significant difference was found between the self-efficacy of labor, state-trait anxiety, and WaC.^[10]^ It was stated that low self-efficacy increases WaC.^[13]^ *P5* and *P352 (theme 7*) felt incompetent to push their baby, also *P168* and *P338 (theme 7,9*) felt incompetent to breastfeed their babies. It was found that young women, who feel worried about their body changes during pregnancy and childbirth, stated more WaC.^[14]^ In our study, *P352 (theme 7*) was afraid of not being able to push her baby because of the put-on weight. It was informed that the WaC affects the women, therefore they question their ability to give birth.^[19]^ Consequently, it is seen that perceiving insufficient labor self-efficacy affects birth worries.^[7,10,13,15–19]^

The results of a study that was performed with 833 women in Belgium and Holland, the women who take midwifery care had lower WaC than those who take standard obstetric care. The results emphasized that the personnel who give antenatal care as an independent model, are important to prevent the WaC.^[9]^ The study of Hildingsson et al^[20]^ reported that 34% of pregnant women had a known midwife during their labor, for which those had more visits for psychological consultation. In addition, they thought that the continuous care was significant, and were glad of the care. Subsequently, 29% of them informed us not have WaC. It was remarked that the severe WaC correlates with perceiving information support inadequately.^[21]^

In our study, it was found that the pregnant women who had been affected by the birthing scenes on TV, birthing stories of their friends and family and on social media, had more labor worries. In addition, *P4*, *P8*, *P11*, *P32*, *P49*, *P131*, *P136*, *P178*, *P215*, *P307*, *P427*, and *P492 (theme 1,6*) stated that they are afraid of thoughts and approaches of other pregnant women and the stories told by the other people. Nilsson and Lundgren^[19]^ determined that previous birth experience is the center of WaC for multipara and these pregnant women had bad experiences with taking care during childbirth. Fenwick et al^[22]^ reported that the negative birth stories cause WaC. Stoll et al noted that WaC, attitudes to the usage of obstetric technology, getting information about pregnancy and the birthing process via social media are significantly correlated with cesarean section.^[23]^ Qiu et al^[13]^ reported that there is a significant correlation between using smartphone applications during pregnancy and more WaC. There is no statistically significant difference between pregnant women who watched and did not watch people/animal births in terms of WaC in our study. However, there is a statistically significant difference among pregnant women who had positive and negative thoughts about animal or human birth. Parallelly to our study, it was found that pregnant women who think social media develops attitudes related to pregnancy and childbirth had more WaC.^[23]^

Although we did not include the pregnant women with risks in our study, 17.3% of pregnant women thought their pregnancy had any risks. There is not a significant difference between the pregnant women who thought and did not think their pregnancy has a risk in terms of WaC in our study. It was reported that pregnant women with risk in their pregnancy have more stress related to pregnancy.^[24]^

As our study was done during the COVID-19 process, there were a few pregnant women stating worries about COVID-19 and those were *P3*, *P70*, *P85*, and *P426 (theme 5*) who were afraid of getting contaminated with COVID-19 and, of the effects of COVID-19 on their baby. In parallel with our study, it was noted that the childbirth worries of pregnant women increased because of Covid-19.^[24]^

One of the most important reasons for fear in pregnancy is labor pain or synthetical oxytocin. *P11*, *P20*, *P27*, *P47*, *P49*, *P51*, *P59*, *P71*, *P86*, *P90*, *P106*, *P173*, *P178*, *P320*, *P339*, *P358*, *P364*, *P374*, *P423*, and *P480 (theme 1*) stated that they are afraid of experiencing the labor pain. Moreover, *P9*, *P46*, *P63*, *P107*, *P113*, *P128*, *P193*, *P333*, *P335*, *P337*, *P397*, *P432*, *P464*, *P474*, *P483*, *P488*, *P511 (theme 2*) notified that they are afraid of experiencing synthetical oxytocin. It was reported that the fears of pregnant women related to having WaC.^[22,25]^

The WaC is affected by attitudes towards hospitals and personnel and previous experiences. *P84*, *P95*, *P126*, *P279*, *P318*, and *P518 (theme 8*) stated that they are afraid of hospital and personnel’s behaviors. It was found that pregnant women who have insecure feelings and attitudes to personnel’s behaviors and the delivery room had more WaC.^[25]^ WaC of pregnant women was found more have negative experiences with healthcare workers.^[19]^ It was reported that a feeling of insecurity about childbirth increases WaC. It was stated that the private emotions that are not solved after previous childbirth and negative experiences affect expectations of childbirth for multiparous.^[22]^ The result of the meta-analysis that was created from the qualitative research about the WaC reported that the lack of confidence makes it difficult to pass to motherhood and the insecure delivery room feeds WaC. This study showed that the necessity to secure care and professional support for reducing the risks and preventing the damages for maternity care is rising.^[26]^ It was informed that the lack of positive expectation which is one of the dimensions of WaC was significantly connected with elective cesarean.^[27]^

It was reported that the women preferred cesarean section because of WaC.^[25]^ In our study, *P262 (theme 7*) stated that she wants to give birth with a cesarean section due to the fear of vaginal delivery. Also, *P27* and *P113 (theme 2*) predicted that they wanted to give birth by taking epidural analgesia. It was reported that women who were terrified of childbirth would prefer to give birth by epidural analgesia or cesarean section.^[23]^ It was noted that the women who stated having severe labor pain among 3189 primiparous or multiparous pregnant women, were more liable to give birth by elective cesarean section.^[27]^ It was found that WaC increased the probability of choice of cesarean section.^[23]^ On the contrary, there are worries about cesarean section. P*7*, *P44*, *P45*, *P48*, *P52*, *P53*, *P64*, *P288*, *P294*, *P342*, *P347*, *P359*, *P395*, *P456*, and *P496 (theme 3*) stated that they did not want to give birth by cesarean section and were terrified of cesarean section or anesthesia. It was noted that while the pregnant women had mid-level WaC, the pregnant women preferring cesarean section had the more intense WaC.^[28]^

The interventions that are made in the labor process cause to increase in WaC.^[26]^ The issues or procedures about childbirth increased WaC.^[25]^ In our study, *P6*, *P95*, *P201*, *P312*, *P365*, and *P391 (theme 4*) indicated their fears were related to examinations or interventions.

The complications in labor or after labor have effects on WaC. In our study, it was stated that *P14*, *P34*, *P103*, *P327*, *P351*, and *P468 (theme 10*) were terrified of bleeding or dying in their labor process and after childbirth. The possibility of tearing of the perineum increased WaC.^[22]^

Although previous birth experience is central to the fear of childbirth for multiparous women,^[22]^ nulliparous have no obscurity about the labor process as those who have a childbirth experience.^[19]^ This can cause increasing WaC. In our study, *P30*, *P56*, *P68*, *P99*, *P133*, *P195*, *P246*, *P291*, *P300*, *P313*, *P390*, *P469*, and *P510 (theme 11*) stated that they had no idea about the labor process and that is why they were terrified. It was noted that the obscurity of the childbirth process enhances the fear of childbirth.^[22]^

As a result, the birth process is affected by women’s physiology, psychology, family, relationships, health systems and providers, and socio-cultural structures. Women expect to be able to manage the birth process, fear, and pain. Worries or anxiety about childbirth is an important obstetric problem that should be managed starting from the antenatal period.^[29–31]^

## 5. Conclusions

The reasons for WaC are multidimensional. The development of WaC begins in the antenatal period. Midwives and other health professionals should contact pregnant women during the antenatal period and prevent the WaC. In addition, those should pave the way for positive posts about birth on both TV and other media channels and prevent negative posts. By managing WaC, positive thoughts about vaginal birth can be spread and the rate of vaginal birth can be increased.

## 6. Limitations

There were some limitations to this study. First, more reasons can be highlighted qualitatively. However, it was not possible in the current study due to the sample size of respondents. Second, the survey only asked about childbirth worries during the antenatal period. To better understand what the reason is, longitudinal studies with questions throughout pregnancy and after childbirth should be conducted.

## Author contributions

**Conceptualization:** Nükhet Kaçar, Rahime Bedir Findik, Özlem Moraloğlu Tekin.

**Investigation:** Nükhet Kaçar, Rahime Bedir Findik, Özlem Moraloğlu Tekin.

**Methodology:** Nükhet Kaçar, Rahime Bedir Findik, Özlem Moraloğlu Tekin.

**Resources:** Nükhet Kaçar, Rahime Bedir Findik.

**Supervision:** Nükhet Kaçar, Rahime Bedir Findik.

**Validation:** Nükhet Kaçar, Rahime Bedir Findik.

**Writing – original draft:** Nükhet Kaçar, Rahime Bedir Findik.

**Writing – review & editing:** Nükhet Kaçar, Rahime Bedir Findik, Özlem Moraloğlu Tekin.
